# Association of Night Shift Work With Chronic Spontaneous Urticaria and Effect Modification by Circadian Dysfunction Among Workers

**DOI:** 10.3389/fpubh.2021.751579

**Published:** 2021-12-02

**Authors:** Yuzhou Huang, Danrong Jing, Juan Su, Zhijun Huang, Han Liu, Juan Tao, Meian He, Xiang Chen, Minxue Shen, Yi Xiao

**Affiliations:** ^1^Department of Dermatology, Xiangya Hospital, Central South University, Changsha, China; ^2^Hunan Engineering Research Center of Skin Health and Disease, Central South University, Changsha, China; ^3^Hunan Key Laboratory of Skin Cancer and Psoriasis, Central South University, Changsha, China; ^4^Center of Clinical Pharmacology, The Third Xiangya Hospital, Central South University, Changsha, China; ^5^Department of Dermatology, Sinopharm Dongfeng General Hospital, Hubei University of Medicine, Shiyan, China; ^6^Department of Dermatology, Tongji Medical College, Union Hospital, Huazhong University of Science and Technology, Wuhan, China; ^7^Ministry of Education Key Laboratory of Environment and Health, School of Public Health, Tongji Medical College, Huazhong University of Science and Technology, Wuhan, China; ^8^National Clinical Research Center for Geriatric Disorders, Changsha, China; ^9^Department of Social Medicine and Health Management, Xiangya School of Public Health, Central South University, Changsha, China

**Keywords:** circadian dysfunction, chronic spontaneous urticaria, effect modification, night shift work, excessive daytime sleepiness

## Abstract

**Purpose:** Night shift work is common in the current working environment and is a risk factor for many diseases. The study aimed to explore the relationship between night shift work with chronic spontaneous urticaria (CSU), and the modification effect of circadian dysfunction on it.

**Methods:** A cross-sectional survey was conducted among Chinese workers. Exposure was measured by night work history and duration. Circadian dysfunction was characterized by excessive daytime sleepiness (EDS). The diagnosis of CSU was made by dermatologists who were investigating on the spot. The effect size was expressed as odds ratios (ORs).

**Results:** A total of 8,057 participants were recruited, and 7,411 (92%) with complete information were included in the final analyses. The prevalence rates of CSU for workers without night shift and those with night shift history were 0.73 and 1.28%, respectively. Compared with workers who never worked night shifts, the risk of CSU increased with the length of night shift work: OR = 1.55 (95% confidence interval [CI]: 0.78–3.06) for duration <5 years and OR = 1.91 (95% CI: 1.12–3.26) for duration ≥5 years. EDS s EDS has been shown to modify this combination. Among workers without EDS, there was no association between night shift and CSU (OR = 0.94; 95% CI: 0.49–1.79). Whereas, in participants with EDS, the correlation was significant (OR = 3.58; 95% CI: 1.14–11.20). However, the effect modification by sleep disturbance was not observed.

**Conclusions:** Night shift work is a risk factor for CSU, and there is a dose-response relationship between night shift work hours and the risk of CSU. This connection may be modified by circadian dysfunction.

## Introduction

Chronic spontaneous urticaria (CSU) is a common allergic skin disorder characterized by wheals or angioedema along with intense itch (1). Although it is usually self-limited and benign, it can cause severe discomfort, lasting from months to years, and rarely represents a serious systemic disease or life-threatening allergic reaction (2). It affects 0.5–1% (1) of the general population, and 0.1–0.3% (3) of children. The burden of urticaria ranked the 5th among all skin conditions according to the 2016 Global Burden of Disease Study. It was estimated that urticaria contributed to 55.49 per 100,000 years loss of healthy life globally (4). The pathophysiology of CSU has not been fully elucidated, but it is clear that the degranulation of mast cells and activation of basophil play the core role in the etiology of urticaria. Previous studies suggest that CSU occurs mostly at night or in the evening with no identifiable triggers, and the severity of cutaneous signs and symptoms is also exacerbated between midnight and morning and shows a significant 24-h rhythm (5–7).

Night shift work is defined as work performed outside of typical daytime work hours. Night shift work is common in the industry to ensure the need for 24-h operation. In industrialized countries, about 20% of the workforce is engaged in shift work (8). Night shift work is a well-established social and biological stress. Previous epidemiological studies suggest that night shift work is a risk factor for obesity (9), diabetes (10, 11), cardiovascular disease (12), breast cancer (13), and mental disorders (14, 15). Night shift work can lead to daily sleep-wake and fasting cycles, and the imbalance of the endogenous circadian timing system, which wildly affects the physiology and behavior, and harmfully influences healthy immune and allergy system.

However, no epidemiologic study has examined the association of night shift work and CSU. In the current study, we conducted a cross-sectional investigation in two groups of workers who frequently worked night shifts. The aim was to investigate the association of night shift work with CSU and to examine the effect modification by circadian dysfunction.

## Materials and Methods

### Study Population

Cross-sectional data from two independent studies were analyzed. The study population comprised of automobile manufacture workers in Shiyan, Hubei who were participants in the Dongfeng-Tongji Cohort Study (16), and non-ferrous metal smelting workers in Hengyang, Hunan who were participants in the Hunan Chronic Disease Cohort Study (17). The Dongfeng-Tongji Cohort was established in 2008 and initially recruited retired workers. The cohort began to recruit in-service workers since 2016. In the current analysis, we used the baseline data collected from the in-service workers. The Hunan Chronic Disease Cohort Health Study was established in 2015 and recruited residents and workers in Hunan rural regions. In the current analysis, we only included participants who were workers to ensure comparability and homogeneity.

### Exposure Assessment

History and duration (years) of rotating night shift work were inquired in the face-to-face interview. Night shift work was defined as “at least three nights per month in addition to working days or evenings.” If a history of the night shift was reported, the cumulative night shift work was then inquired by the investigator. The duration of night shift work was categorized into three groups: never, <5 years, and ≥5 years.

### Outcome Assessment

Diagnosis of skin diseases and inquiry of disease history were performed by certificated dermatologists during the field survey. Clinical manifestation, disease history, and family history of participants were asked, and physical examinations were conducted to diagnose all skin diseases. CSU was diagnosed according to persistent or recurrent typical clinical manifestations of urticaria, with unknown triggers, for more than 6 weeks during the past year.

### Assessment of Covariates

Height and weight were measured by research nurses according to standardized methods. Body mass index (BMI) was calculated as weight (kg)/height^2^ (m^2^). Marital status, socioeconomic status (annual family income and educational level), smoking habits, and passive smoke exposure, and alcohol drinking were inquired by investigators. Anxiety and depression were assessed by the 2-item Generalized Anxiety Disorder (GAD-2) (18) and 2-item Patient Health Questionnaire (PHQ-2) (19), respectively. GAD-2 ≥3 and PHQ-2 ≥3 were the cut-offs for anxiety and depression, respectively. Sleep quality and daytime sleepiness were assessed by the Pittsburgh Sleep Quality Index (PSQI) (20) and Epworth Sleepiness Scale (ESS) (21), respectively. PSQI >5 and ESS >10 were the cut-offs for sleep disturbance and excessive daytime sleepiness (EDS), respectively. History of urticaria was not adjusted since it may cause collider bias in the hypothesized pathway: history of night shift (X) → history of CSU (C_1_) ← genetic susceptibility (C_2_) → current CSU (Y).

### Statistical Analysis

Continuous data were presented as means and standard deviations, and between-group difference was tested using analysis of variance (ANOVA). Categorical data were presented as number (%), and the between-group difference was tested using the chi-square test. A two-level logistic regression model (participant as level-1 unit and study site as a level-2 unit) was used to estimate the association of night shift work with CSU, adjusting for level-1 covariates (age, gender, ethnicity, annual family income, cigarette smoking, alcohol drinking, anxiety, depression) and the random effect (intercept) of study sites. The effect size was presented as odds ratio (OR) and 95% confidence interval (CI). The center effect was examined using the intra-cluster correlation coefficient (ICC). Cubic spline regression was used to examine the potential non-linear association of the duration of night shift work (years) with the prevalence of CSU.

Previous studies have identified associations between circadian and health outcomes (22), and effect modification of associations between night shift work and diseases by circadian and chronotype (11, 23, 24). Therefore, we assessed possible effect modifications of the association between night shift work and CSU by EDS, an indicator of circadian dysfunction. To test for effect modification, we included a multiplicative interaction term in regression models. Stratification analysis by EDS was then conducted if a significant interaction term was identified. In addition, subgroup analysis was conducted by sleep disturbance as determined by PSQI. *P* < 0.05 was considered statistically significant for all tests. Statistical analysis was performed in SAS 9.4 (SAS Institute Inc., Cary, USA).

## Results

**Figure 1:**
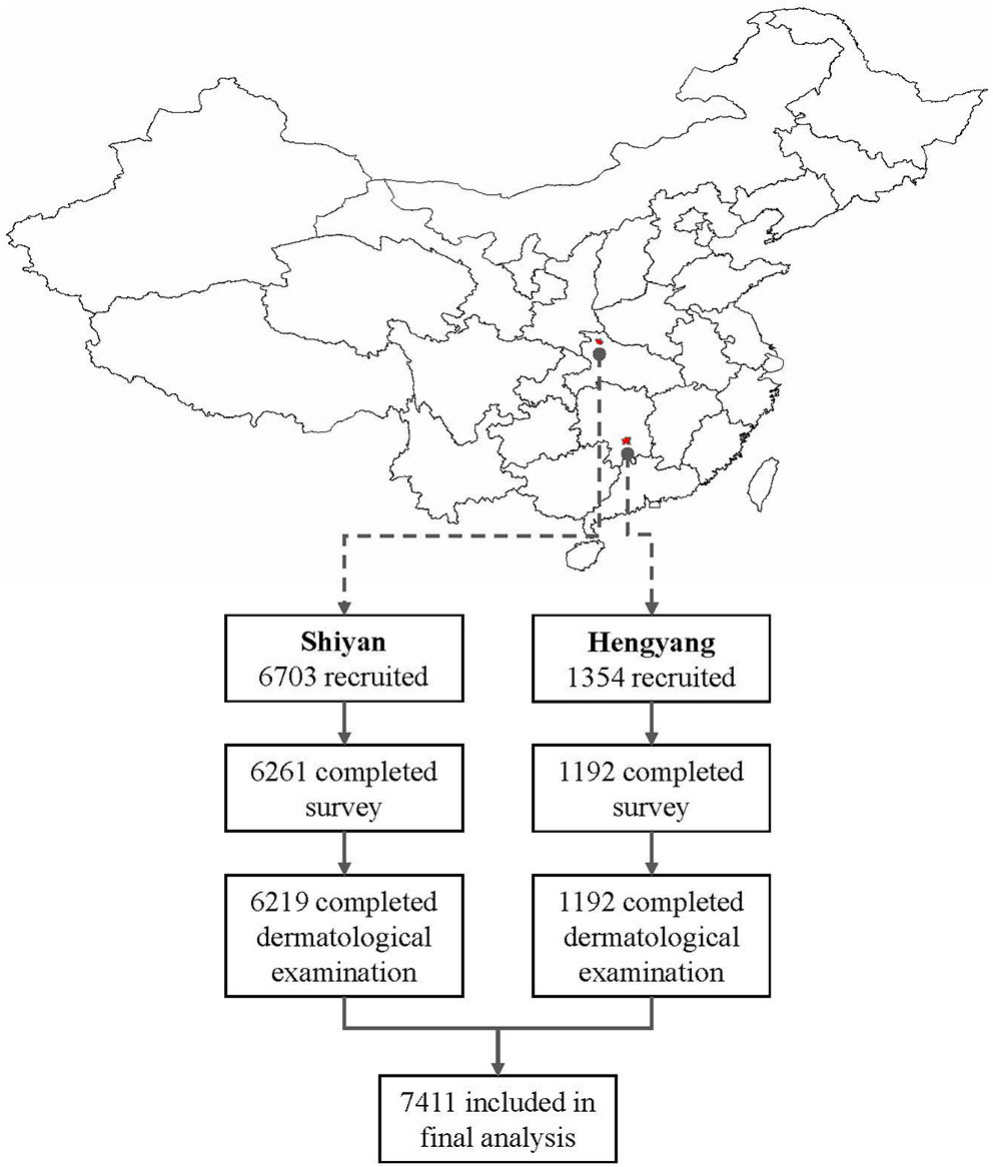
Study sites and flowchart of inclusion of study participants.

A total of 8,057 participants were recruited, and 7,411 (92%) with complete information were included in the final analyses (Figure 1). The mean age was 42.5 ± 8.4 years and 74.9% were male. Comparing the characteristics across the history and duration of night shift work, night shifts were associated with slightly older age, male gender, the Han ethnicity, lower socioeconomic stratum (income and education), smoking behavior, more impaired sleep quality, and more symptoms of depression (Table 1).

**Table 1:** Characteristics of participant by the history and duration of night shift work.

	**History and duration of night shift work**			
**Characteristics**	**Never (***n*** = 2,876)**	** <5 years (***n*** = 1,232)**	**≥5 years (***n*** = 3,303)**	**Ever (***n*** = 4,535)**
**Study site**				
Shiyan	2,762 (96.0)	1,081 (87.7)	2,376 (71.9)	3,457 (76.2)
Hengyang	114 (4.0)	151 (12.3)	927 (28.1)	1,078 (23.8)
Age (years)	41.5 ± 9.1	39.5 ± 9.5	44.4 ± 6.6	43.1 ± 7.8
Male	1,976 (68.7)	984 (79.9)	2,593 (78.5)	3,577 (78.9)
Han ethnicity	2,823 (98.2)	1,217 (98.8)	3,264 (98.8)	4,481 (98.8)
**Marital status**				
Unmarried	345 (12.0)	222 (18.0)	133 (4.0)	355 (7.8)
Married or live together	2,440 (84.9)	960 (77.9)	3,012 (91.2)	3,972 (87.6)
Widowed	15 (0.5)	7 (0.6)	21 (0.6)	28 (0.6)
Divorced	76 (2.6)	43 (3.5)	137 (4.2)	180 (4.0)
**Annual family income (CNY)**				
<30,000	122 (4.3)	94 (7.6)	477 (14.4)	571 (12.6)
30,000–49,999	532 (18.5)	359 (29.1)	1,222 (37.0)	1,581 (34.8)
50,000–99,999	1,324 (46.0)	570 (46.3)	1,343 (40.7)	1,913 (42.2)
≥100,000	898 (31.2)	209 (17.0)	261 (7.9)	470 (10.4)
**Educational level**				
Middle school and below	101 (3.5)	79 (6.4)	468 (14.2)	547 (12.1)
High school	778 (27.1)	497 (40.3)	1,900 (57.5)	2,397 (52.8)
College and above	1,997 (69.4)	656 (53.3)	935 (28.3)	1,591 (35.1)
**Smoking**				
Never	2,000 (69.6)	722 (58.6)	1,656 (50.1)	2,378 (52.4)
Past	145 (5.0)	97 (7.9)	337 (10.2)	434 (9.6)
Current	731 (25.4)	413 (33.5)	1,310 (39.7)	1,723 (38.0)
Passive smoke exposure	1,641 (57.1)	785 (63.7)	2,028 (61.4)	2,813 (62.0)
**Alcohol drinking**				
Never	1,828 (63.5)	724 (58.8)	1,986 (60.1)	2,710 (59.8)
Past	62 (2.2)	53 (4.3)	138 (4.2)	191 (4.2)
Current	986 (34.3)	455 (36.9)	1,179 (35.7)	1,634 (36.0)
BMI (kg/m^2^)	24.0 ± 2.7	24.1 ± 2.9	24.2 ± 2.8	24.1 ± 2.8
Sleep disturbance (PSQI >5)	898 (31.2)	443 (36.0)	1,365 (41.3)	1,808 (39.9)
Daytime sleepiness (ESS >10)	918 (31.9)	426 (34.6)	1,013 (30.7)	1,439 (31.7)
Anxiety (GAD-2 ≥3)	166 (5.8)	77 (6.3)	214 (6.5)	291 (6.4)
Depression (PHQ-2 ≥3)	171 (6.0)	89 (7.2)	285 (8.6)	374 (8.3)

*CNY, Chinese yuan; BMI, body mass index; PSQI, Pittsburgh Sleep Quality Index; ESS, Epworth Sleepiness Scale; GAD-2, 2-item Generalized Anxiety Disorder; PHQ-2:2-item, Patient Health Questionnaire*.

### Prevalence of CSU

The overall prevalence of CSU was 1.07% (79/7,411). The prevalence rates in the two study sites were 1.05% (Shiyan) and 1.17% (Hengyang), respectively, and the center effect of clinical diagnosis was not identified according to the two-level null model (ICC = 0%). In workers who reported no history of night shifts, the prevalence of CSU was 0.73%, while in those who reported a history of night shifts, the prevalence rate was 1.28% (*P* = 0.025). Among subjects who ever worked night shifts, the prevalence rates of CSU were 1.14 and 1.33% in subjects who reported duration <5 years and ≥5 years, respectively (Supplementary Table 1).

### Association of Night Shift Work With CSU

**Figure 2:**
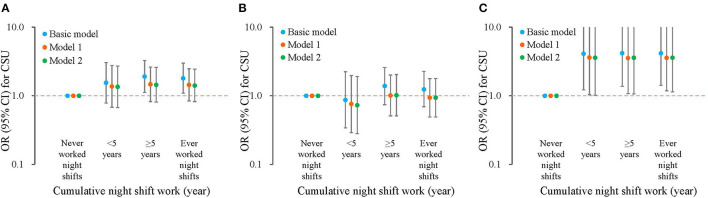
Association of night shift work and chronic spontaneous urticaria, stratified by excessive daytime sleepiness. The basic model was adjusted for age and gender; model 1 was additionally adjusted for ethnicity, marital status, income, educational level; model 2 was additionally adjusted for smoking, passive smoke exposure, alcohol drinking, anxiety, and depression. Logarithmic axis was used for odds ratios. **(A)** All participants; **(B)** participants without EDS; **(C)** participants with EDS. CSU, chronic spontaneous urticaria. EDS, excessive daytime sleepiness; OR, odds ratio; CI, confidence interval.

Night shift work was significantly associated with a higher risk of CSU in a dose-response manner when adjustments were made for age and gender (Figure 2A). Compared with workers who never worked night shifts, the risk increased with the duration of night shift work: OR = 1.55 (95% CI: 0.78–3.06) for duration <5 years and OR = 1.91 (95% CI: 1.12–3.26) for duration ≥5 years. However, when more covariates were included in the models, the result was not statistically significant, although the effect size still indicated a higher risk of CSU (OR = 1.41; 95% CI: 0.82–2.44; *P* = 0.216).

**Figure 3:**
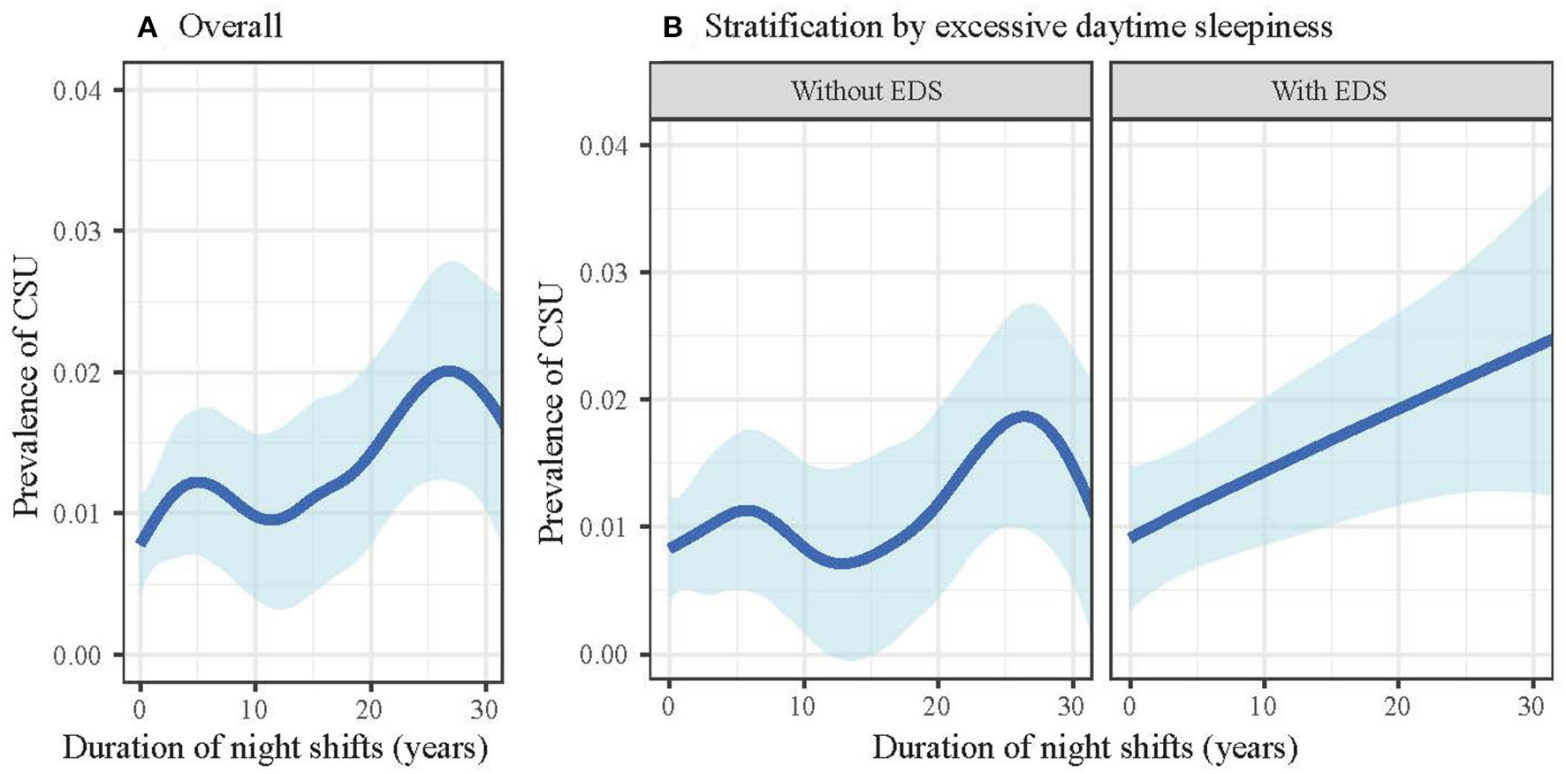
Duration of night shift work and the prevalence of chronic spontaneous urticaria, stratified by excessive daytime sleepiness. The blue curve signifies the estimated prevalence of urticaria, and the light blue band signifies the 95% confidence interval. **(A)** All participants; **(B)** stratification analysis by EDS. CSU, chronic spontaneous urticaria; EDS, excessive daytime sleepiness.

The possible non-linear association of the duration of night shift work as a continuous variable with CSU was examined with the cubic spline. In general, the duration of the night shift was positively associated with CSU, but variations could be observed (Figure 3A).

### Effect Modification

A multiplicative interaction term between EDS and night shift work was then included in regression models. After adjustments, a significant interaction term was identified (β = 1.33, *P* = 0.032), indicating a modification effect. Stratification analysis by EDS was then conducted (Supplementary Table 1). In participants without EDS, the effect size of night shift work was close to null (OR = 0.94; 95% CI: 0.49–1.79; *P* = 0.841) (Figure 2B). In contrast, in workers with EDS, night shift work was significantly associated with CSU (OR = 3.58; 95% CI: 1.14–11.20; *P* = 0.029) after full adjustments with a greater effect size (Figure 2C).

Similarly, when treating the duration of night shift as a continuous variable, the association of night shift with CSU was an irregular curve in subjects without EDS; while in those with EDS, night shift was almost linearly associated with CSU (Figure 3B). We also examined the effect of modification by sleep disturbance. However, the associations of night shift work with CSU were consistent in two subgroups (Supplementary Figure 1).

## Discussion

This cross-sectional study investigated the association of night shift work with CSU among Chinese workers. Unique effect modification by daytime alertness was identified, leading to differential associations of night shift work with CSU. That is, in workers with specific circadian dysfunction manifested as daytime sleepiness, the night shift was a risk factor for CSU with strong effect size, while in those with normal daytime alertness, the effect of night shift diminished substantially.

We identified a correlation between night shift work and CSU, and the risk of CSU increased with the duration of night shift work compared to workers who have never worked overnight. Since the early 1960's, many reports have confirmed the importance of circadian rhythm in allergic diseases (25, 26). Allergy-related peripheral clocks (e.g., mast cell clocks) have been considered as significant drivers for rhythmic allergic reactions. Recent studies have shown that abnormal light/dark environments that mimic jet lag can exacerbate viral-induced asthma-like inflammation. When studying the relationship between night work and food allergies, nurses who participated in night shifts had a higher incidence of food allergies than nurses worked as a regular shift schedule (27). Collectively, these studies suggest that mismatched environmental zeitgeber arrivals may exacerbate allergic reactions.

Urticaria is caused by the release of histamine and other inflammatory mediators from mast cells and basophils mediated by immunoglobulin E- and non-immunoglobulin E. (2). CSU is an endogenous disease that is closely related to autoimmunity, especially the immunoglobulin G (IgG) antibody to the alpha subunit of the IgE receptor, which is seen in 35–40% of patients. Basophils and cutaneous mast cells can be activated, leading to a late-phase-like perivascular infiltration about small venules and hive formation (28). Several *in vivo* studies demonstrated that circadian rhythms drive daily rhythms in IgE/mast cell-mediated allergic reactions. It was reported that wild-type mice had a 24-h time-dependent change in passive skin allergic reaction and passive systemic allergic reaction of IgE/mast cell-dependent allergic reactions (29). Similarly, this time-dependent change also did not occur in mice undergoing mechanical disruption of the central suprachiasmatic nucleus clock or in mice undergoing adrenalectomy (30, 31). These findings suggest that the circadian clock plays a crucial role in the production of daily rhythms of IgE/mast cell-mediated allergic reactions (32).

Based on the stratification analysis, we found that EDS showed a modification effect on this association. While no study or report that directly support our epidemiological finding, a genome-wide association study identified a total of 42 loci and genes to be associated with EDS (33). Among the genes involving EDS reported by Wang et al., four genes [DOCK1 (34), ERBB4 (35), SLC39A8 (36), and CACNA1C (37)] were reported to be associated with allergic reactions in previous studies. Among them, DOCK1 (34), ERBB4 (35), and SLC39A8 (36) were reported to be related to asthma, and CACNA1C (37) was proved to impact the prognosis of CSU. Despite the lack of direct evidence, we speculate that night shift may result in certain epigenetic changes on the genes that link EDS and allergy. This hypothesis needs further investigation.

### Limitations and Strengths

The study was the first to investigate the association of night work with CSU. The study has several strengths. First, we introduced several covariates that might confound the association of night shift with CSU, including income, education, smoking, alcohol, depression, and EDS. Second, the sample size of the study was relatively large, and night shift work was common among workers; this enables us to investigate the association with sufficient power of a statistical test. Third, this was a population-based study, and the Berkson bias was minimized compared to hospital-based studies.

The primary limitation of the study is that no conclusion on the causal relationship can be drawn owing to the cross-sectional design. Second, the study population was workers; this may limit the external validity of the findings and the generalizability to populations. Last but not least, workers might be exposed to complex occupational factors that were not observed in our study. For example, the level of physical activity of workers is higher than that of the general population (38), and they may be exposed to occupational risks including heavy metals, environmental pollutants, and noise at work (39). Nevertheless, the dose and type of occupational exposures are not likely to be altered by the timing of work.

In summary, we identified a link between night shift work and CSU. Circadian dysfunction might modify the association of night work with CSU. Future research may require more delicate assessment on the exposure to night shift work as well as longitudinal observations on its effect on the incident CSU. This study has clinical implication for dermatologists and primary care physicians with respect to the treatment and management of CSU. The study also provides new evidences for mechanism studies.

## Data Availability Statement

The raw data supporting the conclusions of this article will be made available by the authors, without undue reservation.

## Ethics Statement

The studies involving human participants were reviewed and approved by this study was conducted according to the guidelines laid down in the Declaration of Helsinki. All procedures involving study participants were approved by the Institutional Research Ethics Boards of Xiangya School of Public Health, Central South University (approve# XYGW-2016-10), School of Public Health, Tongji Medical College, Huazhong University of Science and Technology (approve# 2016-IEC-S128), and Hubei University of Medicine (approve# 2016-74-41). Written informed consent was obtained from all participants before the investigation. The patients/participants provided their written informed consent to participate in this study.

## Author Contributions

ZH, MH, XC, and MS designed the study. MS and DJ analyzed the data. YX and YH drafted the manuscript. YH, DJ, JS, ZH, HL, JT, MH, and XC interpreted the data and critically revised the manuscript. JS and MH obtained the funding. All authors participated in the field survey, data collection, and gave final approval to the version submitted for publication.

## Funding

This work was supported by the National Key Research and Development Project of China Precision Medicine Initiative (2016YFC0900802) and the Program of Introducing Talents of Discipline to Universities (111 Project, No. B20017).

## Conflict of Interest

The authors declare that the research was conducted in the absence of any commercial or financial relationships that could be construed as a potential conflict of interest.

## Publisher's Note

All claims expressed in this article are solely those of the authors and do not necessarily represent those of their affiliated organizations, or those of the publisher, the editors and the reviewers. Any product that may be evaluated in this article, or claim that may be made by its manufacturer, is not guaranteed or endorsed by the publisher.
