# Supplementary material for: Association of Night Shift Work With Chronic Spontaneous Urticaria and Effect Modification by Circadian Dysfunction Among Workers
Source: Front Public Health. 2021 Dec 2;9:751579. doi: 10.3389/fpubh.2021.751579 (PMC8674304; doi:10.3389/fpubh.2021.751579)
Supplement: Supplementary file 1 [file Data_Sheet_1.PDF]

Table S1. Association of rotating night shift work with chronic spontaneous urticaria, stratified by excessive daytime sleepiness

		Basic model [a]		Model 1 [b]		Model 2 [c]	
Stratum	Cases/participants (%)	OR (95% CI)	*P*	OR (95% CI)	*P*	OR (95% CI)	*P*
Overall							
Never worked night shifts	21/2876 (0.73)	1 (reference)		1 (reference)		1 (reference)	
<5 years	14/1232 (1.14)	1.55 (0.78–3.06)	0.213	1.37 (0.68–2.76)	0.373	1.35 (0.67–2.71)	0.407
≥5 years	44/3303 (1.33)	1.91 (1.12–3.26)	0.017	1.47 (0.82–2.62)	0.193	1.44 (0.81–2.59)	0.216
Ever worked night shifts	58/4535 (1.28)	1.80 (1.09–2.99)	0.048	1.44 (0.84–2.48)	0.190	1.41 (0.82–2.44)	0.216
Without EDS							
Never worked night shifts	17/1958 (0.87)	1 (reference)		1 (reference)		1 (reference)	
<5 years	6/806 (0.74)	0.87 (0.34–2.24)	0.779	0.76 (0.29–1.97)	0.564	0.73 (0.28–1.91)	0.521
≥5 years	26/2290 (1.14)	1.39 (0.74–2.58)	0.306	1.01 (0.51–2.00)	0.981	1.02 (0.51–2.04)	0.948
Ever worked night shifts	32/3096 (1.03)	1.24 (0.69–2.26)	0.473	0.94 (0.49–1.78)	0.840	0.94 (0.49–1.79)	0.841
With EDS							
Never worked night shifts	4/918 (0.44)	1 (reference)		1 (reference)		1 (reference)	
<5 years	8/426 (1.88)	4.09 (1.22–13.74)	0.023	3.61 (1.04–12.54)	0.044	3.58 (1.02–12.57)	0.046
≥5 years	18/1013 (1.78)	4.18 (1.37–12.76)	0.012	3.54 (1.07–11.73)	0.039	3.58 (1.06–12.06)	0.040
Ever worked night shifts	26/1439 (1.81)	4.15 (1.42–12.08)	0.009	3.57 (1.15–11.04)	0.027	3.58 (1.14–11.20)	0.029

EDS: excessive daytime sleepiness. OR: odds ratio. CI: confidence interval.

[a] Adjusted for age and gender.

[b] Additionally adjusted for ethnicity, marital status, income, educational level.

[c] Additionally adjusted for smoking, passive smoke exposure, alcohol drinking, anxiety, and depression.

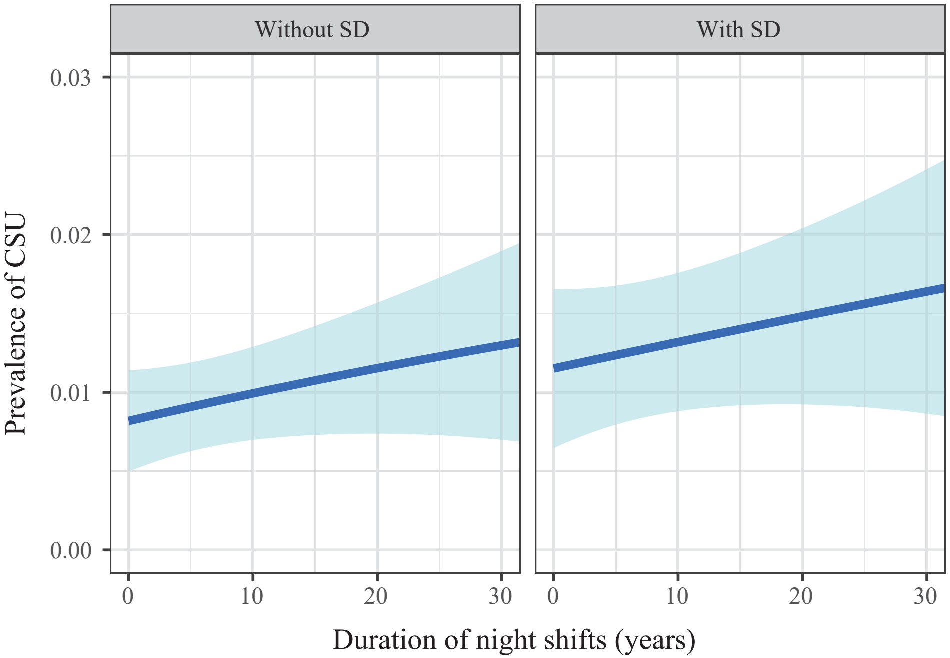

Figure S1. Duration of night shift work and the prevalence of chronic spontaneous urticaria, stratified by sleep disturbance. The blue curve signifies the estimated prevalence of urticaria, and the light blue band signifies its 95% confidence interval. (A) All participants; (B) stratification analysis by EDS. CSU: chronic spontaneous urticaria. SD: sleep disturbance.
